# Improving the visualization and detection of tissue folds in whole slide images through color enhancement

**DOI:** 10.4103/2153-3539.73320

**Published:** 2010-11-29

**Authors:** Pinky A. Bautista, Yukako Yagi

**Affiliations:** Department of Pathology, Massachusetts General Hospital, Harvard Medical School, Boston MA, 02114

**Keywords:** Digital pathology, enhancement, image analysis, image quality, luminance, saturation, tissue fold detection, virtual slide, visualization, whole slide imaging

## Abstract

**Objective:**

The objective of this paper is to improve the visualization and detection of tissue folds, which are prominent among tissue slides, from the pre-scan image of a whole slide image by introducing a color enhancement method that enables the differentiation between fold and non-fold image pixels.

**Method:**

The weighted difference between the color saturation and luminance of the image pixels is used as shifting factor to the original RGB color of the image.

**Results:**

Application of the enhancement method to hematoxylin and eosin (H&E) stained images improves the visualization of tissue folds regardless of the colorimetric variations in the images. Detection of tissue folds after application of the enhancement also improves but the presence of nuclei, which are also stained dark like the folds, was found to sometimes affect the detection accuracy.

**Conclusion:**

The presence of tissue artifacts could affect the quality of whole slide images, especially that whole slide scanners select the focus points from the pre-scan image wherein the artifacts are indistinguishable from real tissue area. We have a presented in this paper an enhancement scheme that improves the visualization and detection of tissue folds from pre-scan images. Since the method works on the simulated pre-scan images its integration to the actual whole slide imaging process should also be possible.

## INTRODUCTION

Digital imaging has been found to be useful in virtually all of medical fields. In recent years, the introduction of high resolution, automated whole slide imaging has enabled pathologists to conveniently view and browse digital versions of glass slides on computer monitors and across computer networks - a task that used to require the direct examination of the physical slide, locally through a microscope.[1–3] Digital images allow the development of digital algorithms for tissue analysis,[4–7] hence are obvious candidates for computational analysis. The practical application of multispectral and hyper-spectral imaging to pathology has also attracted the attention of several researchers, particularly its usefulness in bringing out details that are otherwise inconspicuous with the conventional RGB color imaging.[8–10]

In order for whole slide imaging to be fully utilized in the clinical (and research) setting, one of the important issues that needs to be tackled is the consistency of the image quality. As was discussed by Yagi and Gilbertson,[11] the digital image quality can be adversely affected by tissue processing artifacts, such as tissue folds, originating from histology laboratory. To capture high-resolution, whole slide images at high speed, many whole slide imaging devices use low resolution, snap-shot, “pre-scan” image prior to high-resolution digitization. The pre-scan image is used to: (1) identify the location of tissue sections on the slide and (2) select focus points on the slide for auto-focusing. The number of auto-focus points varies by specimen and device, but tends to range from tens to hundreds. The data generated from these auto-focused points is used to guide the working distance during high-resolution, high-speed scan. The selection of auto-focusing points is therefore critical to the focus and quality of the whole slide image.

The digital algorithm involved in the selection of optimum focus points should be carefully designed. Tissue areas affected by artifacts, such as tissue folds or air bubbles, have different focus depths compared to normal tissue areas. Hence when a focus point is selected from the affected areas, the quality of the scan in the neighboring areas degrades, i.e. becomes blurred. In the case of tissue folds whose color histogram overlaps with that of the tissue itself, there is a high probability that the scanner may actually select focus points on top of them.

Image artifacts can also have an adverse effect on the image segmentation results. Spatial or morphology filtering are the popular approaches to minimize the segmentation errors due to artifacts.[12–17] For image analysis in pathology, the architectural, textural, and morphological patterns of the tissue components are exploited to delineate the true tissue area from the image artifacts. On the assumption that cells are regularly distributed, Guesebroek[13] proposed a distance graph algorithm to identify regions of interest, while errors caused by tissue artifacts were corrected by deletion operations. The utilization of the texture and morphology patterns of cells as features was also explored by Karacali and Tozeren[17] to locate regions of interest. Statistical classifier and clustering algorithms using the unique staining patterns of the tissue components as feature variables are also popular approach to differentiate tissue structures from the background.[14,16,17] Petushi *et al*.[16] converted the original RGB color representation of the H&E stained image pixels to CIELab color representation, and the regions of interest, i.e. chromatin rich and stromal region, were identified by clustering the a and b chromaticity components in the CIELab color space. Moreover, thresholding and clustering the difference between the color saturation and luminance of the pixels were utilized by Palokangas[14] to segment the tissue folds to exclude them from the image analysis results of an H&E stained image.

Color enhancement while it improves the visual feel of an image can also serve as a pre-processing step for the detection and segmentation of an object of interest. For medical images, color enhancement can be a very valuable tool to visualize, detect, or segment specific structures. Several published papers addressed the enhancement of medical images from different imaging modalities. To enhance the structures in endoscopic images, Ohyama *et al*,[18] proposed the Laplacian color enhancement. Retinal images suffer non-uniform illumination and hence Gopal and Jayanthi[19] introduced a way to enhance retinal images by considering the geometry of the retina. Color enhancement techniques that apply spatial transforms in conjunction with color transforms are also being employed.[20,21]

The objective of this paper is to improve the visualization and detection of tissue folds from low- pixel resolution images (pre-scan or thumbnail images) so that this information can be used to avoid tissue folds in the whole slide imaging auto-focusing process and thereby improving the quality of high-pixel resolution whole slide images.[22] We present and discuss a color enhancement method, which ^]^ we initially proposed[22], which gives preferential emphasis on tissue folds. In that method, the weighted difference between color saturation and luminance of the image pixels was used as shifting factor to the original RGB color values of the pixels. The physical basis on using the luminance and saturation for the detection of tissue folds in low-resolution images is that the amount of dye that the tissue can absorb is a function of its thickness. Tissue folds, being thicker than immediately the surrounding area, absorb more dye and therefore appear darker (lower luminance) and express stronger color saturation compared to adjacent non-folded areas. The proposed enhancement method can be integrated to the software-driven processes involved in scanning the glass slides to produce better quality images. It can also serve as a pre-processing step to further improve the quantification of nuclei area, and the segmentation of other related tissue structures.

## MATERIALS AND METHODS

### Tissue Sections and Slides

Twelve, de-identified, H&E stained slides were received from the histology laboratory at the Massachusetts General Hospital. The slides had been cut manually, stained, and cover-slipped by an automated device. They represented a range of tissues including breast, liver, and esophagus.

### Imaging System

Two different whole slide imaging systems were used to scan the H&E stained tissue slides. One was the NDP (Nanozoomer Digital Pathology) whole slide scanner (Olympus, USA) and the other was the DX40 scanner (Dmetrix Inc., Tucson, AZ, USA). These systems can scan in color (RGB) mode at a spatial resolution of 0.50 *μ*m/pixel for the Dmetrix40 and 0.420 *μ*m/pixel for the NDP scanner at an optical magnification of 20×. Both scanners also have features for manual or automatic selection of tissue areas. While the selection of focus points (vide supra, introduction) is always done automatically in the case of the DX40 system, the NDP system allows the option of user selection of focus points (from low resolution, pre-scan image) or automatic, machine-defined focus points. In our experiment the focus points were machine-defined, since our concern was to evaluate the effectiveness of the proposed tissue-fold enhancement algorithm.

### Whole Slide Images

The images were scanned by our laboratory technical staff or by pathology residents rotating in the laboratory. Our aim was to evaluate the ability of the proposed enhancement scheme to identify tissue folds in low resolution, pre-scan images (so that the technique could be used, eventually, by whole slide images to identify the location of tissue folds prior to a high resolution, whole slide scan). However, we did not have access to the actual raw pre-scan images from the scanners in this experiment. Therefore, as proxy to the true pre-scan images, we used the lowest digital resolution available through the viewer software available through each of the devices. For the NDP viewer, these were JPEG images sampled by the NDP software to a display resolution of typically 20 *μ*m/pixel. For the DX40, these were TIFF images at typically 10 *μ*m/pixel. We do not believe that this is a limitation on the results of the study since whole slide imagers use different approaches and formats for their low-resolution pre-scan images.

### Color Enhancement

One of the effective ways to increase the color contrast of an image, while maintaining its hue, is to transform the original RGB color of the image to HSV (hue, saturation, value) color space and modify only the saturation or luminance component of its pixel.[23] The relation between RGB and HSV color spaces is defined as follows:

(1) $$H=\;\cos^{-1\;}\beta$$

$$\mathrm{wherer}\;\beta \;=\;\left\{\frac{\frac{1}{2}\left[\left(R\;-\;G\right)+\left(R\;-\;B\right)\right]}{\sqrt{{\left(R\;-G\right)}^{2\;\;}+\;\left(R\;-B\right)\left(G\;-\;B\right)}}\right\}$$

(2) $$S\;=\;1-\frac{3}{R+G+B}\left[\min\left(R,\;G,\;B\right)\right]$$

(3) $$V\;=\;\frac{1}{3}\left(R+G+B\right)$$

**Figure 1:**
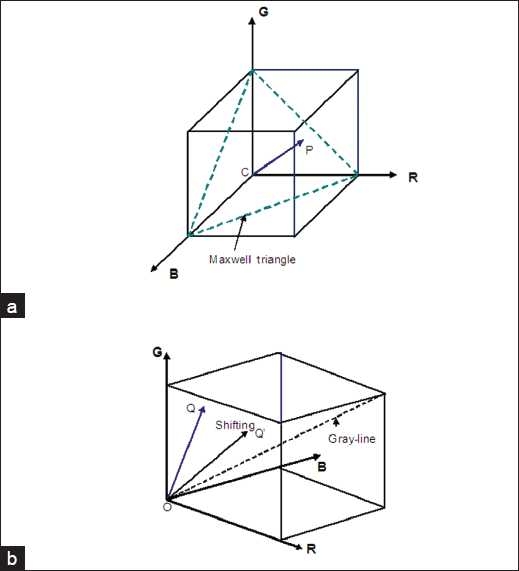
Illustrations on the effect of color shifting and scaling[23]. (a) The RGB color cube illustrating the effect of scaling to the luminance of the image. The length of vector CP is related to the luminance of the image pixel and the triangle with vertices at the maximum values of the R, G, and B axes is called the Maxwell triangle. Scaling the RGB values increases or decreases the length of the vector which would in turn vary the luminance of the image pixel; (b) An illustration on the effect of shifting. While shifting the original color vector OQ to OQ’ reduces the saturation of the image pixel, shifting the vector OQ farther from the gray line and closer to the RGB cube increases the saturation of the pixel. Hence the saturation of an image can be manipulated by shifting the color vector by an appropriate amount

Since the HSV color components share very weak correlation, we can manipulate one of the color components by one of several techniques that are commonly applied to gray-level enhancement processes without necessarily affecting the other components. The saturation and luminance enhancement is conventionally done by undertaking forward and reverse color transformation between RGB and HSV color spaces. In the forward color transformation the original RGB colors of the pixels are converted into their HSV color equivalent, i.e. RGB to HSV, where the color saturation or luminance of the image pixels is independently modified. The reverse color transformation, i.e. HSV to RGB, is then undertaken to view the effect of the modification (enhanced image). However, through scaling and shifting[23] these forward and reverse color transformations can be bypassed. Consider a pixel’s luminance that is represented by the length of the vector CP in Figure 1a. The luminance of the image can be directly modified by scaling the vector CP. Furthermore, shifting the vector OQ in Figure 1b closer or away from the gray line will reduce or increase the saturation of the image. That is, moving the vector OQ closer to the gray line will reduce the image color saturation and moving the vector away from the gray line and closer to the RGB cube boundary increases the saturation of the pixel. Scaling and shifting therefore can effectively enhance the luminance and saturation of the image without necessarily undertaking the forward and reverse color transformations, i.e. RGB to HSV then HSV to RGB.

Let us consider f to denote the RGB color vector of an image I, f= (*f*_1,_ *f*_2,_ *f*_3_)^T^ where *f*_1,_ *f*_2,_ *f*_3_ correspond to the red, green, and blue pixel values, i.e. 0≤*f*_k_ ≤1 *k*=1,2,3 (1=red, 2=green, 3=blue) then scaling and shifting of the original RGB color components can be expressed by equations 4 and 5, respectively:

(4) f’ = γf

(5) f’ = f + σ,

where the constants γ and σ denote the scaling and shifting factors, respectively. While scaling changes the luminance of the image, shifting, on the other hand, changes the saturation of the image. Since in this example the color pixels are provided with the same scaling or shifting factor, the image hue is unchanged. The scaling and shifting factors can also be represented by any suitable functions:

(6) $$f_{k}=\;\gamma \left(f_{k}\right)f_{k\;}k=\;1,2,3$$

(7) $$f{’}_{k}=\;f_{k}+Ó\left(f_{k}\right)\;k=\;1,2,3$$

where *γ*(*f*_k_) and Ó(*f* _k_), respectively, denote the scaling and shifting functions. However, since these scaling and shifting functions do not necessarily result to the same value for all *k*=1,2, 3 the red, green, and blue color components of a pixel could be modified at different degrees, thus changing the hue of the image.[24]

### Enhancement of Tissue Folds

We employed shifting to enhance the presence of tissue folds. In this case, our main concern lay in the appropriate shifting function that would minimize the changes in hue while accentuating the spectral color of the folds. To satisfy this condition, the gray level value of a pixel at each color channel should be shifted by the same amount. Because of the thickness of tissue folds they generally exhibit higher saturation and lower luminance compared to normal tissue areas such that by taking the difference between the saturation and luminance components of the pixel as shifting factor, the colorimetric attributes of tissue folds can be accentuated. Taking this into consideration, equation (7) can be expressed as:

(8) $$f{’}_{k}=\;f_{k}+Ó\left(f_{\;\mathrm{sv}}\right)\;k=1,2,3,$$

where *f* _sv_ = S(*x,y*) – V(*x,y*) and S(*x,y*) and V(*x,y*) correspond to the pixel’s color saturation and luminance at location x, y, respectively. Here, pixels at different spatial locations experience differing color shifts depending on the nature of the tissue structure to which the pixel belongs. Moreover, the polarity of the shift can be either negative or positive. It is negative when the pixel’s color saturation is lower than its luminance, e.g. white areas; and positive when the pixel’s color saturation is higher than its luminance, e.g. tissue folds. Apparently it is the difference between the saturation and luminance, *f* _sv_, which controls the polarity of the shifts. To further modify the color saturation of the image pixels we introduced a constant to vary the magnitude of the shifting factor in equation 8:

(9) $$f{’}_{k}=\;f_{k}+\alpha_{k}Ó\left(f_{\;\mathrm{sv}}\right)\;k=\;1,2,3$$

where α*_k_* ∈ 
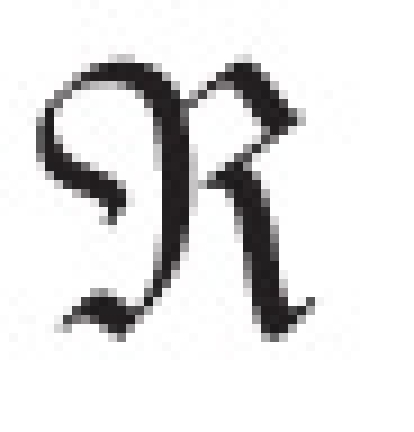
 and α*_1_* = α*_2_* = α*_3_* = α.

Let us examine how the enhancement formulation in equation (9) affects the color saturation of an image pixel. Neglecting the *x, y* location of the pixels for simplification of expression, the new color saturation can be expressed as follows:

(10) $$S’\;=\;1-\frac{3}{R+G+B+3\alpha \left(S-V\right)}\;\left[\min\left(R+\alpha \left(S-V\right),G+\alpha \left(S-V\right),B+\alpha \left(S-V\right)\right)\right]$$

$$\begin{array}{l} =\{1-3\frac{R+\alpha \left(S-V\right)}{R+G+B+3\alpha \left(S-V\right)}\;\mathrm{if}\;R\;\mathrm{is}\;\min\;1-3\frac{G+\alpha \left(S-V\right)}{R+G+B+3\alpha \left(S-V\right)}\;\mathrm{if}\;G\;\mathrm{is}\;\min\;\;1-3\frac{B+\alpha \left(S-V\right)}{R+G+B+3\alpha \left(S-V\right)}\;\mathrm{if}\;B\;\mathrm{is}\;\min\; \end{array}$$

Let us represent the change in saturation by the following:

(11) ∆V = S'−S

If *R* is the minimum then,

(12) $$\begin{array}{l} \mathrm{ÄS}\;=\left(1-3\frac{R+\alpha \left(S-V\right)}{R+G+B+3\alpha \left(S-V\right)}\right)-\left(1-3\frac{R}{R+G+B}\right)\;=\frac{3\left(2R-G-B\right)\alpha \left(S-V\right)}{\left(R+G+B\right)\left(R+G+B+3\alpha \left(S-V\right)\right)} \end{array}$$

The same derivation can be undertaken when *G* or *B* is the minimum. Since the term (2*R–G–B*), or (2*G–R–B*) as in the case when *G* is the minimum, or (2*B–R–G*) when *B* is the minimum is always negative, it is the product between the enhancement coefficient α and σ(*f*_sv_) that commands the change in the color saturation. If the product is greater than zero the saturation decreases or vice versa. On the other hand, we can easily derive the change in luminance i.e. Δ*V=V´–V* from equation (3) :

(13) $$\mathrm{∆V}\;=\;\alpha \left(S-V\right)$$

This shows that while saturation decreases when α>*0* and (S–V)>*0* the luminance increases, which implies that an increase in the color saturation correspondingly decreases the luminance of an image pixel, or vice versa.

**Figure 2:**
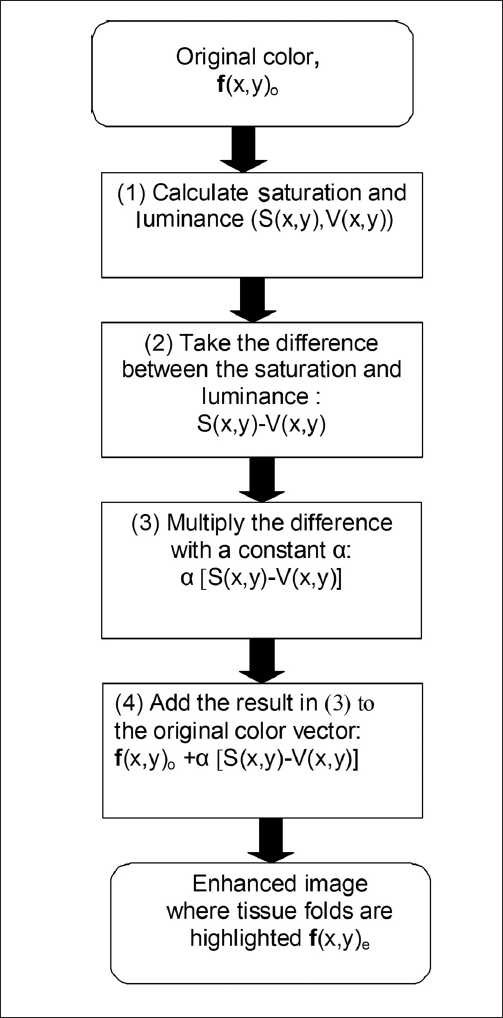
Block diagram of the enhancement procedure to detect tissue folds. First the color vector of an image pixel is accessed and its saturation and luminance values are calculated. Then the difference between the saturation and luminance is weighted and added to the original color vector. The process is repeated for all image pixels to produce an enhanced image where tissue folds are highlighted

The diagram in Figure 2 illustrates the enhancement procedure to detect tissue folds in whole slide images. In the diagram *f*(*x, y*)_o_ corresponds to the vector representing the original R, G, and B color values of an image pixel at locations *x, y*, and *f*(*x, y*)_e_ to the enhanced color values; S(*x, y*) and V(*x, y*) are the corresponding color saturation and luminance values of the image pixel at locations *x, y*, respectively. The color saturation and luminance of the pixel is first calculated, and then the difference between them is weighted and added to the original RGB color values of the pixel.

## RESULTS

### Color Attributes of the Digital Slides

**Figure 3:**
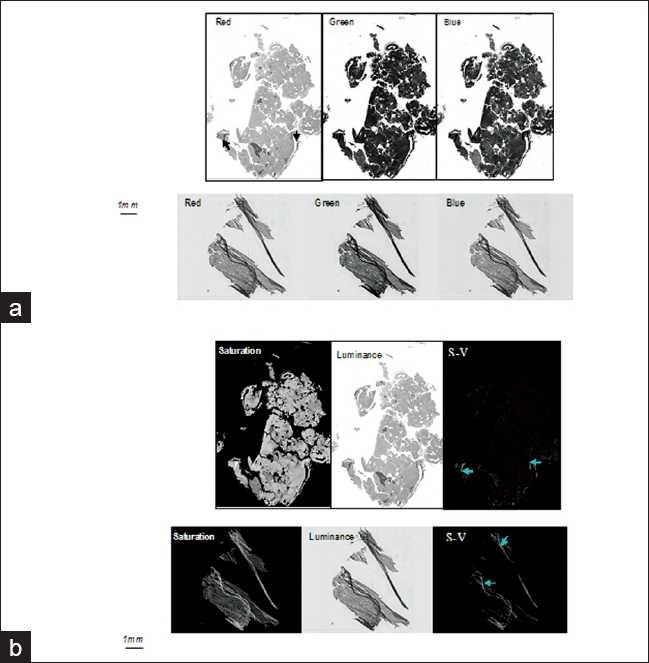
Effect of staining variations to the RGB, and saturation and luminance color components of whole slide images.(a) RGB channel images of two whole slides, i.e. red, green, and blue channel images. The image in the upper row was scanned by the DX40 Dmetrix scanner and the image in the lower row was scanned by the NDP scanner. The varying colorimetric attributes of the scanned images may cause difficulty in specifying the effective color channel for fold detection/segmentation; (b) Saturation and luminance components of the RGB color image and the gray-level presentation of the difference between the saturation the luminance images where the dark areas correspond to areas in the original image in which the saturation of the pixel is less than its luminance

Panel A in Figure 3 shows the RGB color images of two tissue slides, which are decomposed into their red, green, and blue channel images. These images demonstrate that there is no consistent color channel from which tissue folds can be extracted, especially when variations in staining exist between the slides. A transformation of an RGB image to another color space would allow us to examine the color attributes of an object from different color perspectives. Panel B in Figure 3 displays the color saturation and luminance components of the images after implementing the color transformation from RGB to HSV color space. We can observe that regardless of the colorimetric variation between the images, their color saturation and luminance share similar tendencies: (i) tissue areas occupied by tissue folds appear more saturated compared to other tissue areas; and (ii) the luminance of folded areas is relatively lower with respect to other tissue areas. Moreover, the tissue-fold areas are distinctly marked after taking the difference between the color saturation and luminance components of the image pixels despite the color variations that exist between the images.

### S-V Histograms of the Tissue Folds and Non-folds

**Figure 4:**
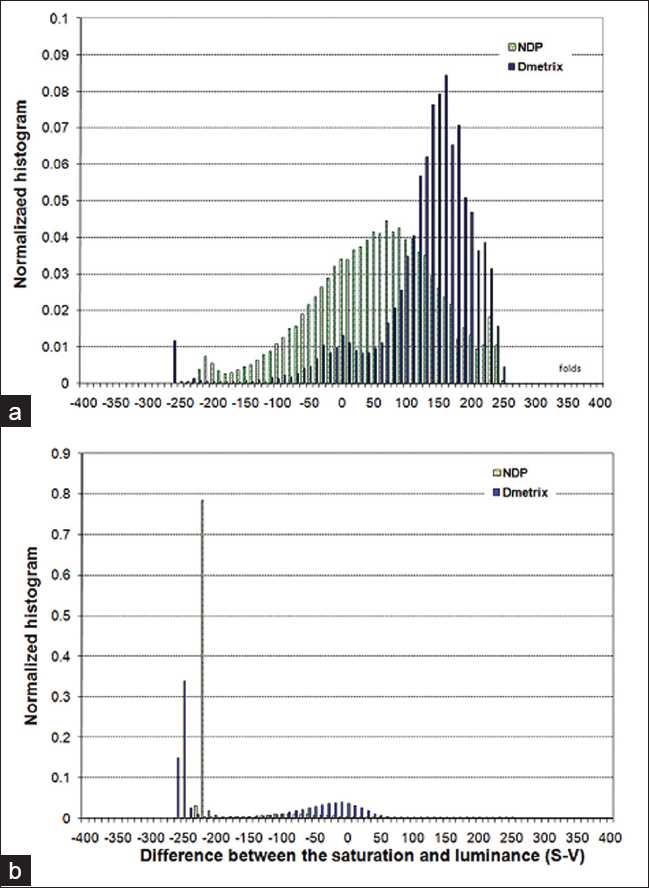
Histograms of the S-V of tissue folds and non-tissue folds of the images shown in Figure 8. (a) Histogram of the manually segmented tissue folds. We can see that although the tissue folds were segmented from slides having differing staining conditions and were scanned using different scanners they showed similar tendencies - folds tend to acquire positive S-V values. The pixels that acquired negative S-V values in the plot could correspond to pixels that belong to patches of non-folded areas which were not carefully delineated by the manual segmenter. (b) Histogram of non-folded areas. In contrast to folded areas, the non-folded areas are more inclined to acquire negative values. The pixels with positive S-V values could correspond to nuclei areas, since like tissue folds nuclei are also stained darker compared to other tissue components

Figure 4 displays the normalized S-V histograms of the manually labeled tissue fold and non-tissue fold areas of the two H&E stained images, which were scanned by the NDP and Dmetrix scanners, shown in Figure 8. These histograms reveal that regardless of the image or slide condition, i.e. spectral color variations or staining differences, fold pixels are more inclined to acquire positive S-V values compared to non-fold pixels. The negative S-V of the fold pixels as shown in the histogram in Figure 4a represents the small white patches within the fold regions that were not carefully differentiated in the manual segmentation. Likewise the pixels with positive S-V in the histogram of the non-fold pixels could represent the hematoxylin-stained nuclei pixels, since like the tissue folds they are also stained darker compared to eosin-stained tissue structures.

### Determination of the Value of α

**Figure 5:**
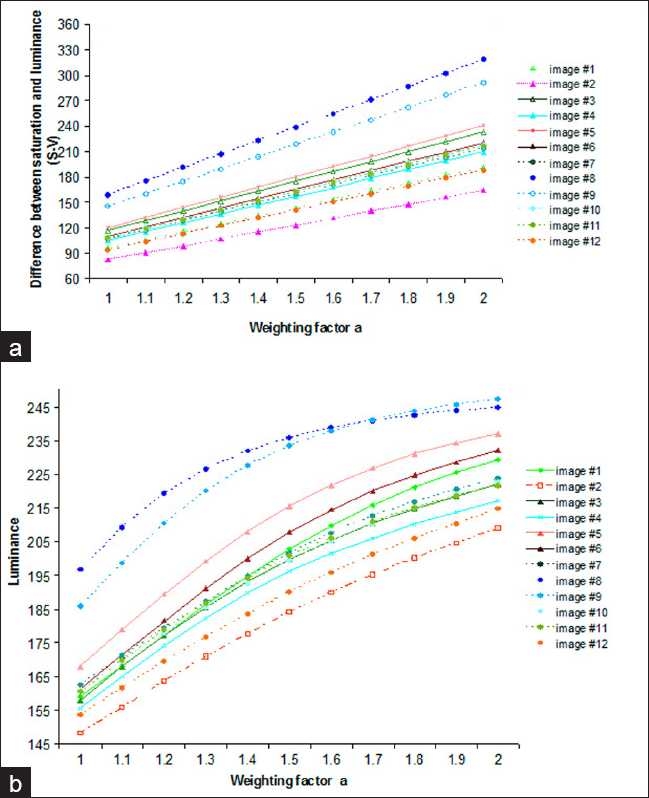
Plots showing the variations in the difference between the saturation and luminance, i.e. S-V and the luminance (V) of the fold pixels against different values of the enhancement coefficient, α. Each plot was produced by taking the average of 500 1×1 pixel fold samples for each image. (a) Plot illustrating the tendencies of the S-V values for the two set of images, i.e. scanned by Dmetrix and that of NDP scanner, for different values of α. (b) Plot demonstrating the effect on the luminance of the fold pixels for different values of α. It is observed from this plot that the luminance of the pixels do not significantly vary beyond α =1.5

Determining the effective value of α is important in improving the visualization and detection of folds. Even if the difference between S and V of the tissue fold samples increases linearly with α as illustrated in Figure 5a, but for most of them their corresponding enhanced luminance values do not have a significant increase starting from α =1.5, Figure 5b. Hence with respect to the image samples that were used in our experiment, the value α = 1.5 can be considered as an optimum value to emphasize the tissue folds.

### Enhancement of the Tissue Folds

**Figure 6:**
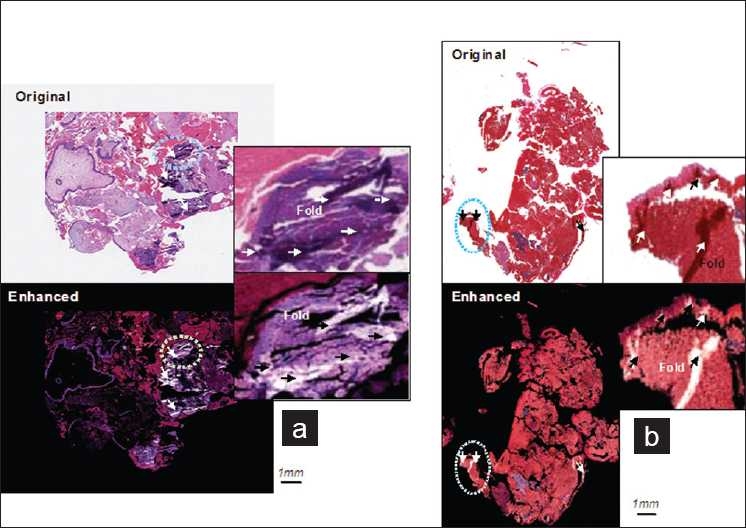
Enhancement results. (a) Results for NDP scanned images; (b) Results for Dmetrix DX40 scanned images. The solid white areas in the enhanced images are associated to tissue folds. The tissue folds which appear occluded in the original image since they share close colorimetric attributes to their neighboring areas are better emphasized in the enhanced images. The folds that are indicated by the arrows in the magnified enhanced images further show how tissue folds become well differentiated after application of the enhancement

In Figure 6 the resulting enhanced images by setting α to 1.5 are shown wherein the solid white areas correspond to tissue folds. Comparing these images to their originals we can see that tissue folds that were originally obscured are now better emphasized. To further investigate the result of the enhancement, a magnified view is facilitated for selected tissue areas that contain folds. From the magnified images we can observe that the colorimetric difference between fold and non-fold areas has been improved after we applied the proposed enhancement. The tissue folds, which are indicated by arrows, can now be distinctly identified from other tissue components.

### Color Variations Between Whole Slide Scanners

**Figure 7:**
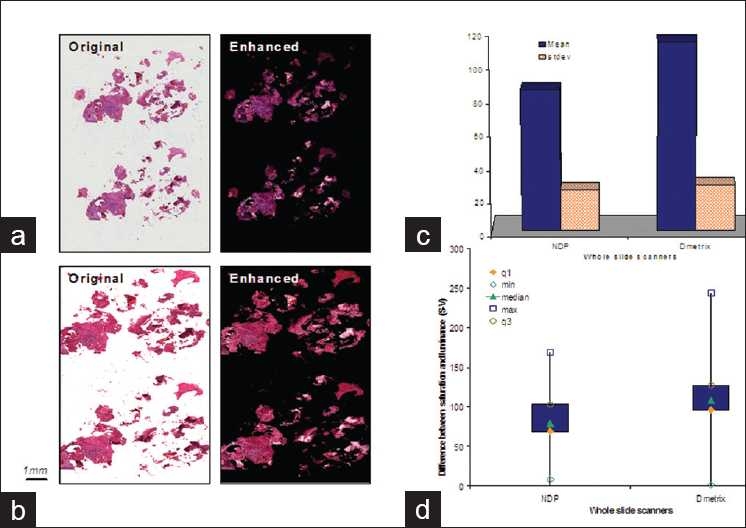
Original and enhanced Images of the same tissue slide scanned by the two scanners used in the experiment, i.e. Dmetrix and NDP whole slide scanners, and their corresponding S-V statistics. (a) Scanned by NDP scanner; (b) Scanned by Dmetrix scanner; (c) Plot showing the mean and the standard deviations; (d) other statistics demonstrated by a box plot. It is noted that while the same slide was scanned by both machines the images acquired different colorimetric attributes. However, despite the difference in the color attributes in the scanned images, the folds are still highlighted in the enhanced images. The mean of the calculated S-V samples show small discrepancy between the NDP and Dmetrix samples although the images were produced by scanning the same slide. Also the maximum S-V value is higher for the image scanned by the Dmetrix scanner which can imply that images scanned by Dmetrix scanner exhibit relatively higher saturation

Staining variations or differences in the color calibrations among scanning devices could both result to color variations in the tissue components and in the tissue folds. We scanned the same tissue slide using the NDP and the Dmetrix whole slide scanners to constrain the cause of the color variations to the scanners’ color calibration settings. The difference in the color calibrations between the two whole slide scanners is clearly demonstrated by the images in Figures 7a and 7b. We examined the statistics of 500 representative fold samples taken from the NDP and Dmetrix scanned images shown in Figures 7a and 7b. The plots in Figures 7c and 7d indicate that the color saturation setting for the Dmetrix scanner is relatively higher compared to the NDP scanner. Since the variation tendencies that we observed in the S-V values in the plots are similar to the variations observed in the original 12 images, Figure 3, we used the same value of α, i.e. α=1.5, to highlight the tissue-fold areas. The emphasized tissue-fold areas are demonstrated by the images in the second column of Figures 7a and 7b.

### Detection of Tissue Folds

The results presented in the previous section demonstrate the viability of the proposed enhancement scheme to highlight the presence of tissue folds. To determine how the enhancement method fares in localizing tissue folds, we performed automatic and manual detections independently and compared the results. In the manual detection we labeled the folds based on the RGB attributes of the pixels, while in the automatic detection the difference between the enhanced and original luminance of the image pixels was utilized as numerical feature:

(14) $$\mathrm{dV}\;=\;V_{e}-V_{o}$$

where V_e_ is the luminance after enhancement and V_o_ corresponds to the original luminance (before enhancement) of the image pixel. The parameter dV is deemed to be greater than zero for pixels that belong to fold areas such that:

(15) $$\begin{array}{l} \{\underset{\mathrm{not}\;\mathrm{fold}\;\mathrm{otherwise}}{\mathrm{fold}\;\mathrm{dV}\;>\;0} \end{array}$$

**Figure 8:**
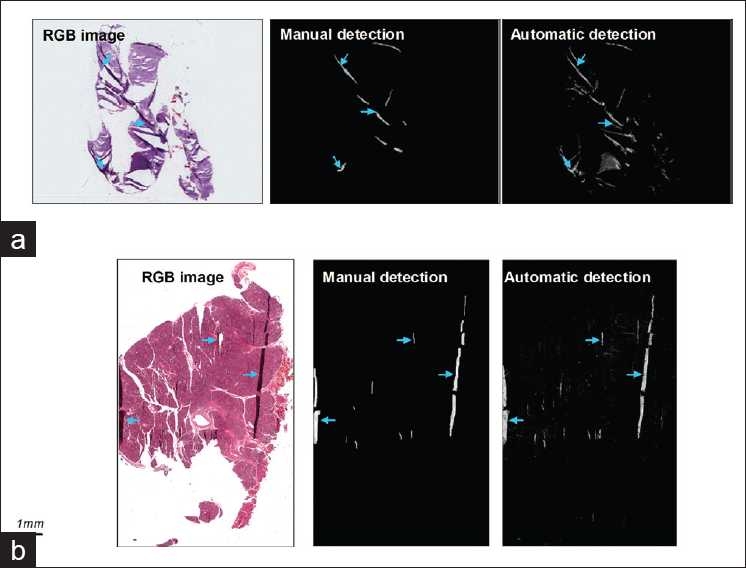
Fold detection results. Column 1 represents the original RGB image. Column 2 represents the saturation of the pixels that were detected manually and Column 3 shows the gray level representation of the difference in the luminance, i.e. V_e_– V_o_, in the automatic detection. (a) Detection results showing some areas that were not successfully detected manually but were detected automatically. (b) Detection results showing some mislabeling of tissue pixels in the automatic detection result. These mislabeled pixels are believed to belong to nuclei as these tissue components exhibit comparable saturation characteristics to the folds

We evaluated the fold detection results for 28 slides in which case 16 more slides were added to the 12 original slides from the same scanners. Table 1 shows the overlap ratio between the manual and automatic segmentation results where we can see that the ratio of some images is not as high as the other images. This can be explained by considering the segmentation results shown in Figure 8. In Figure 8a, folds which occupy smaller areas and folds whose color is similar to the neighboring tissue structures were not successfully detected with manual detection but were detected with automatic detection. Although folds could be successfully detected by applying equation (15), mislabeling of pixels could sometimes occur as illustrated by the resulting image in Figure 8b. Thus the variations in the overlap ratio can be accounted to: (i) undetected tissue-fold areas in the manual detection were detected in the automatic detection; or (ii) mislabeling of pixels.

**Table 1:** Overlap ratio between results of manual and automatic segmentation. While in the manual detection, folds were identified by referring to the RGB color image of the tissue slide, the numerical feature calculated from the difference between the luminance of the enhanced and original images served as features in the automatic fold detection. The low values of the overlap ratio might have been caused by mislabeling of image pixels or undetected tissue folds in the manual segmentation

sample	Overlap ratio TP/(FN+TP+FP)	sample	Overlap ratio TP/(FN+TP+FP)
1	0.3277	15	0.4133
2	0.5977	16	0.1971
3	0.5553	17	0.6017
4	0.3009	18	0.3553
5	0.1151	19	0.3497
6	0.1960	20	0.3718
7	0.0616	21	0.5304
8	0.1025	22	0.5475
9	0.1924	23	0.2189
10	0.0617	24	0.4506
11	0.3065	25	0.4306
12	0.2106	26	0.5238
13	0.6746	27	0.6536
14	0.2397	28	0.7791

TN –True negative; FN- False Negative; FP-False positive; TP-True negative

### Application to Other Stained Images

**Figure 9:**
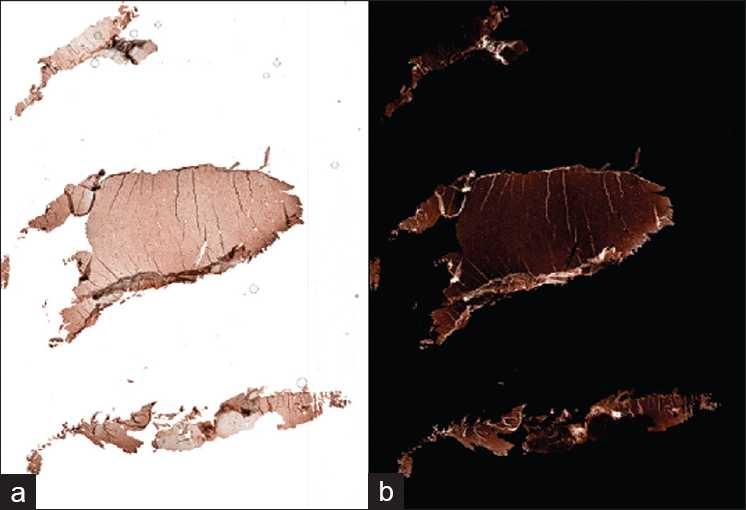
Enhancement results for IHC stained slides. (a) Original RGB color image; (b) Enhanced image; the folds are indicated by arrows

The current method can also be applied to tissue images other than H&E stained images such as immunohistochemical (IHC) stained images with hematoxylin counter stain. We applied the present enhancement scheme to a liver tissue slide stained with FOXP3, Figure 9a, and we found the effective value of α to be 1.2 for tissue folds to be clearly delineated from other tissue structures.The result shows, Figure 9b, that as long as the current assumption holds true, i.e. tissue folds have higher color saturation than its luminance and are more saturated compared to other tissue areas, similar results can also be produced for tissue slides stained with other types of stain. When the colorimetric characteristics of tissue folds deviate from the present assumption, i.e. folds appear brighter rather than darker compared to other tissue structures, the tissue folds may not be properly highlighted with the current enhancement scheme.

## DISCUSSION

Tissue artifacts occur during the preparation of the histopathology slides and although measures are undertaken to reduce their occurrence, they still are common among pathology laboratories. These artifacts might not greatly matter to pathologists viewing the slides directly under a microscope, but when these slides become subject for whole slide scanning and especially when further digital image analysis is undertaken, the presence of these artifacts is not desirable. In fact according to the initial investigation done by Yagi and Gilbertson,[11] the presence of tissue artifacts, including folds, can impact the quality of whole slide images. Although common among tissue slides the detection of tissue artifacts such as folds is not yet popularly addressed, especially detecting the folds from pre-scan image of the whole slide image. Segmentation of tissue folds from high resolution *n*×*m* images sampled from *N×M* whole slide images however was considered by Palokangas *et al*.[14] by clustering the color pixels using the k-means clustering algorithm. The main drawback of the method is its inability to detect the presence or absence of tissue folds before applying the segmentation by pixel clustering. The enhancement method introduced herein can be incorporated to further improve their segmentation method.

Differences in colorimetric attributes in whole slide images can be due to either difference in the stained slide itself (histological parameters) or to the parameters of the digitization (imaging) process (we will ignore downstream parameters such as compression or monitor quality which are outside the scope of this paper). However, for images created on the same scanner that has been appropriately calibrated and using the same acquisition parameters, variations in imaging context can be minimized and the differences can be solely attributed to histological parameters, the most important of which is the staining condition. Staining can be affected by a number of factors including the tissue itself, the thickness of the tissue section, the length of time at which tissue is exposed to stains, etc.

### Limitations and Future Works

Since the color saturation and luminance of a pixel is independent from its hue, consistent results were still achieved even when differences in staining conditions exist among tissue slides. In the technique discussed in this paper, the weighting factor α (which modulates the importance of saturation and luminance in the detection of folds) plays an important role to the efficient delineation between fold and non-fold areas. In the experiment, α was set to 1.5 for H&E stained slides; however, when IHC slides (with light hematoxylin counter-stain) were examined, it appeared that the most effective value for α seemed to be closer to 1.2. This likely has to do with the strength of the counterstain.

Automated use of the current technique not only successfully detects large folds clearly visible to the human eye, but also objective folds that are inconspicuous and which manual methods fail to detect. Application of the technique however did result, in some cases, in the mislabeling of isolated pixels as tissue folds. This is best seen in the third column of Figure 8b. We believe these pixels represent large, strongly staining nuclei. This is possible since nuclei often stain significantly darker than surrounding areas and thus exhibit the same luminance and saturation characteristics as the folds. To remove these errors (to improve specificity) we have to either re-design the detection algorithm, for example integrating a spatial filter to minimize the mislabeling of nuclei, or modify the assignment of the enhancement coefficients such as assigning different coefficients to the saturation and luminance and adjusting them in an independent manner to allow for effective delineation between folds and nuclei.

## CONCLUSION

Histology laboratory artifacts can affect the quality of a whole slide image in a variety of ways. One of these involves tissue folds and their effect in focusing algorithms. In this paper we have addressed the detection of tissue folds from the pre-scan image of a whole slide image by proposing an enhancement method that adaptively shifts the original RGB color of the image by an amount equivalent to the difference between its saturation and luminance components. Since the enhancement method works well on the simulated pre-scan images, its integration to the actual whole slide imaging process should be possible.
